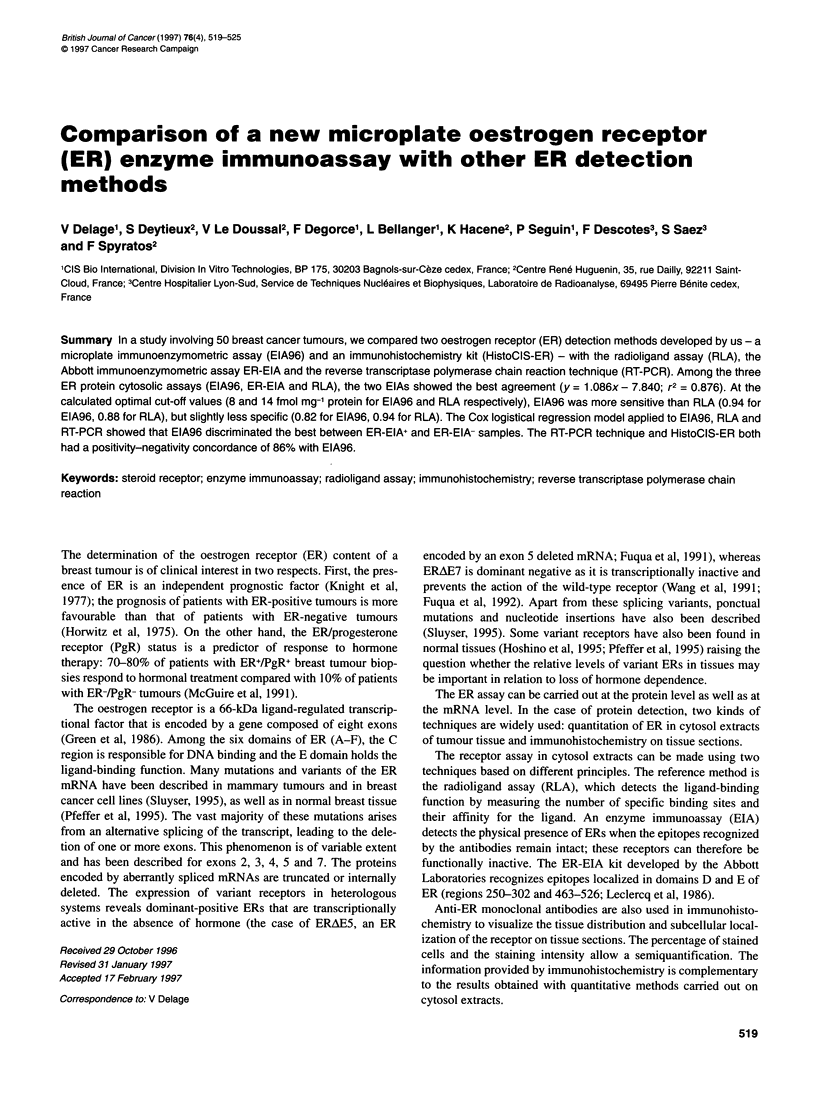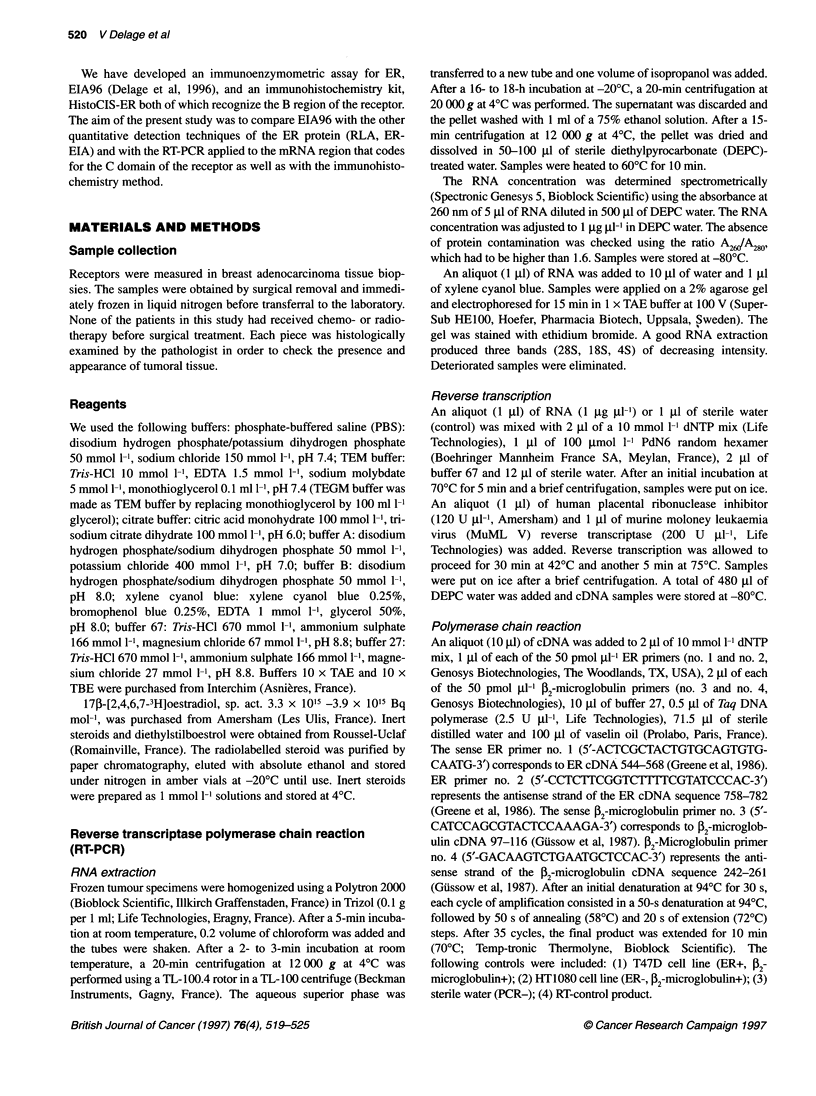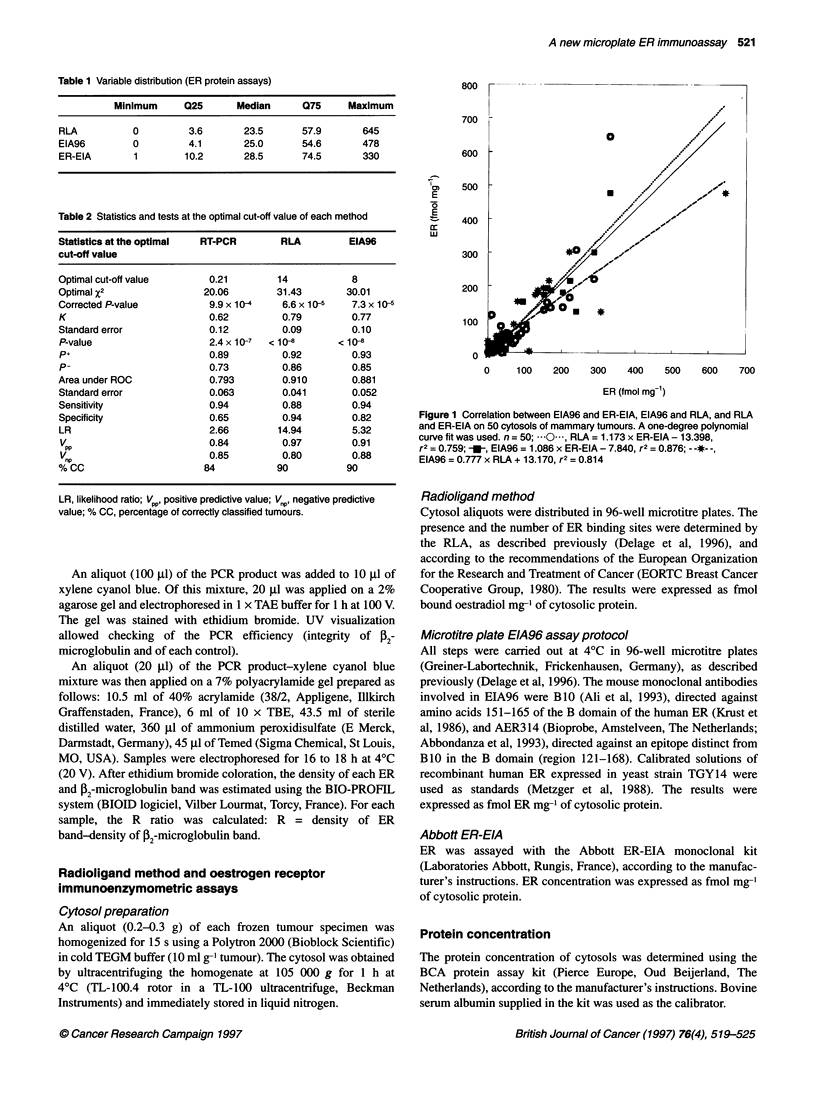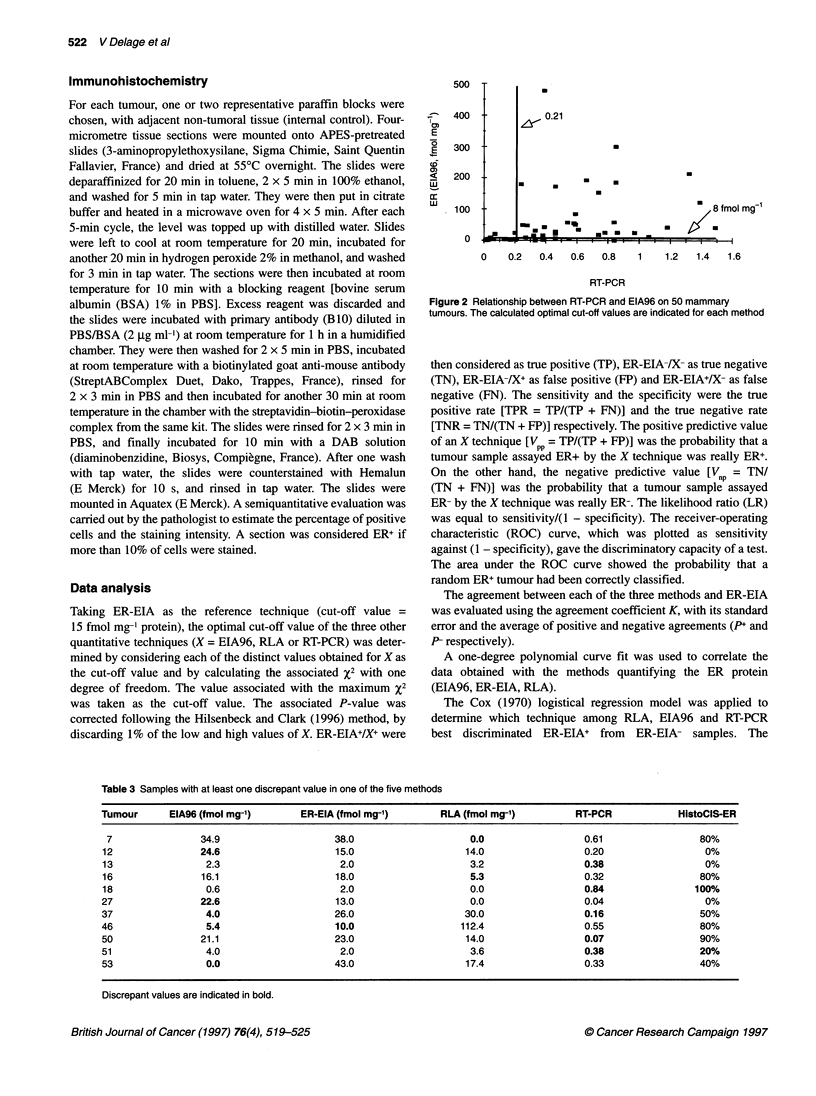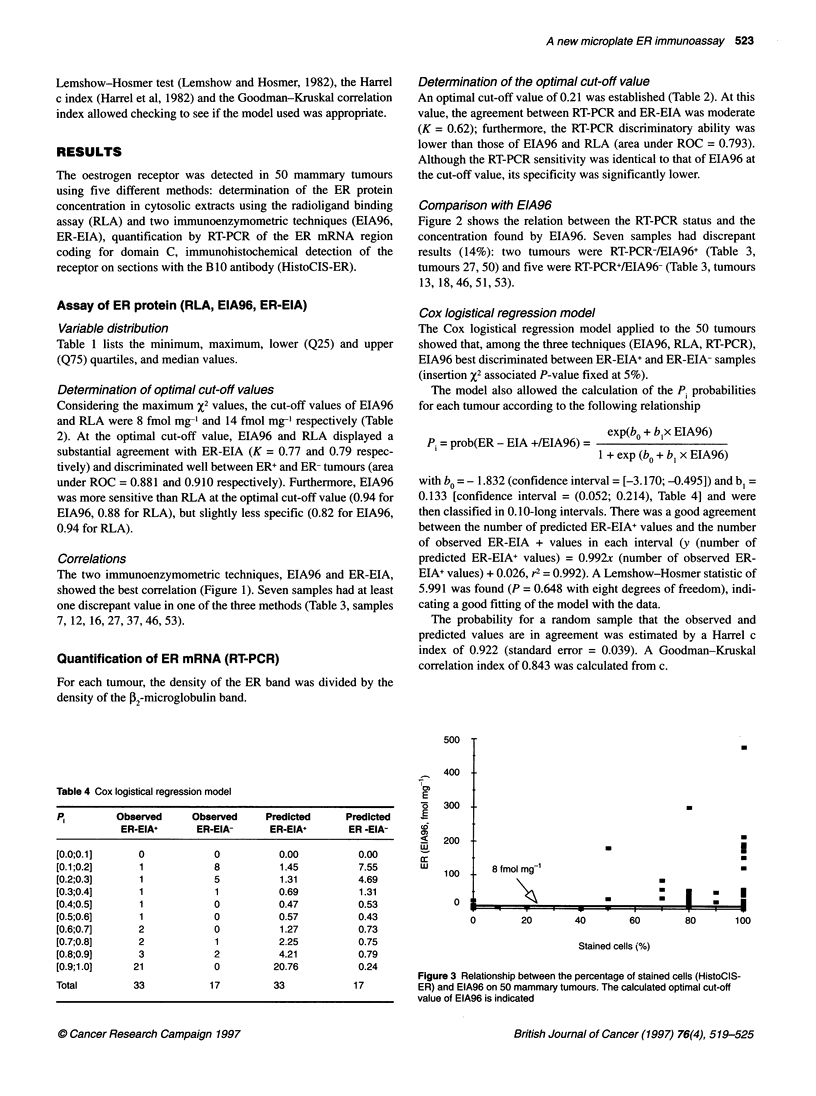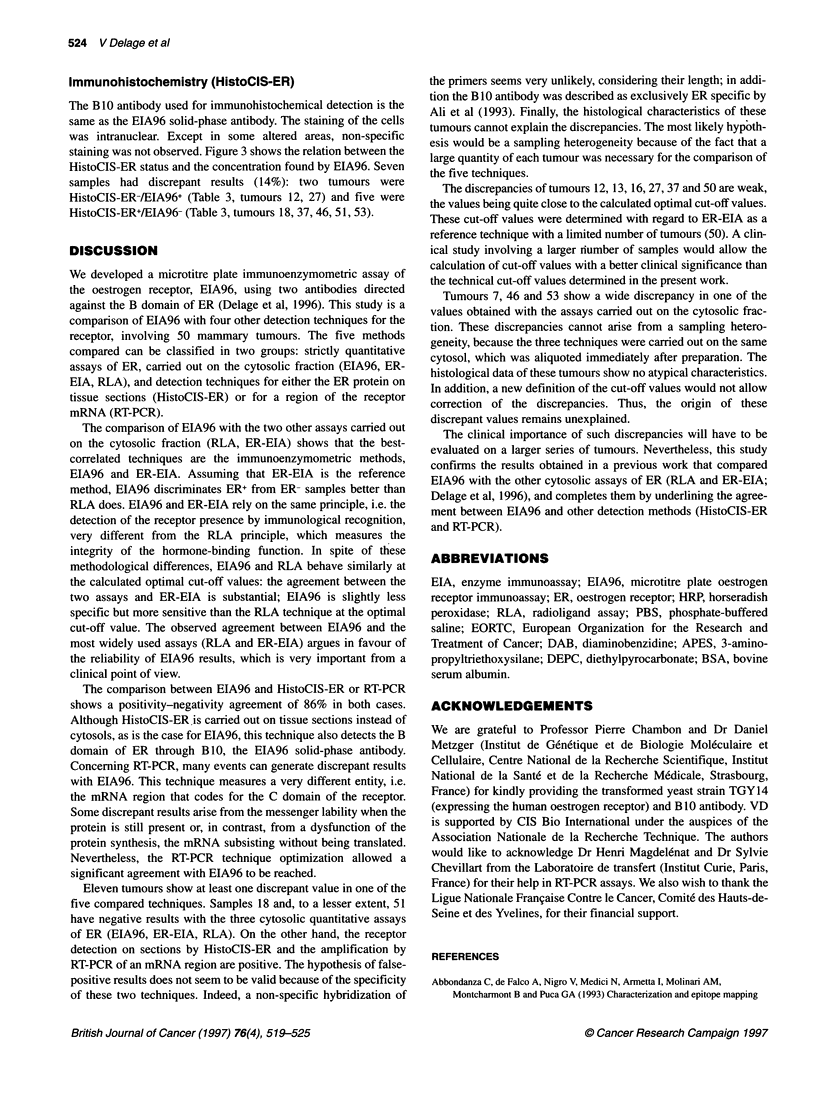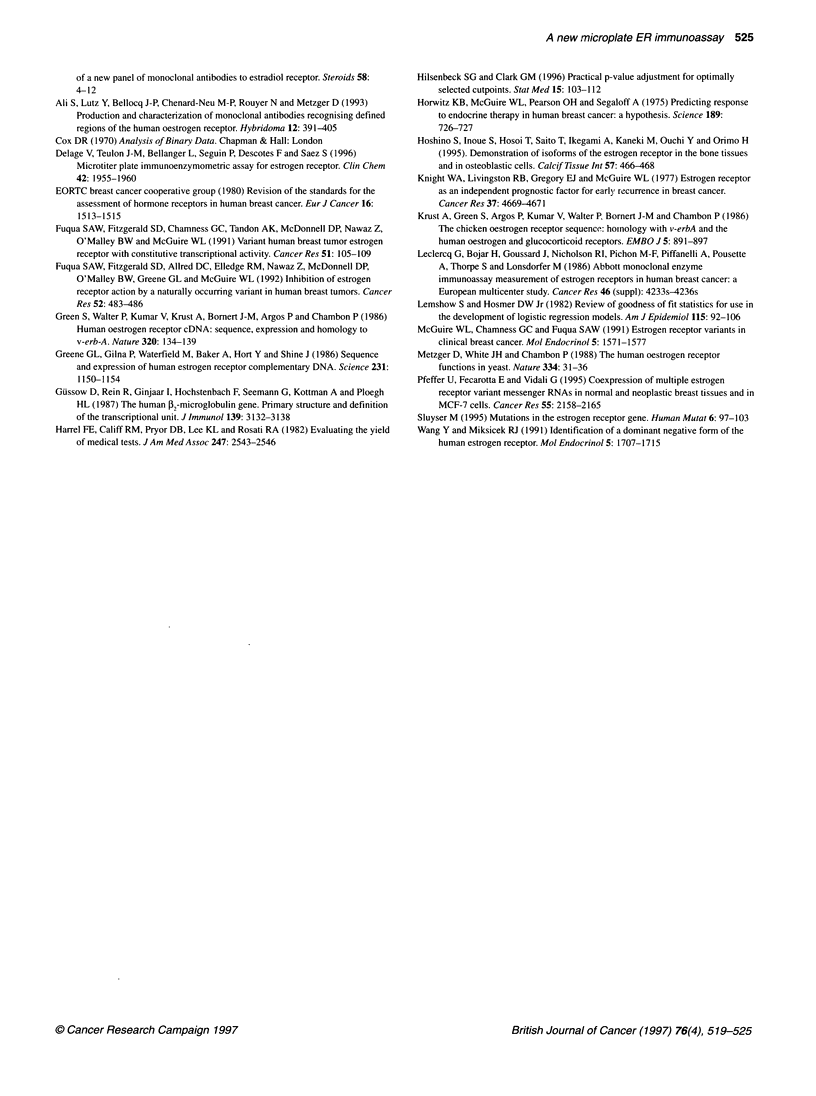# Comparison of a new microplate oestrogen receptor (ER) enzyme immunoassay with other ER detection methods.

**DOI:** 10.1038/bjc.1997.418

**Published:** 1997

**Authors:** V. Delage, S. Deytieux, V. Le Doussal, F. Degorce, L. Bellanger, K. Hacene, P. Seguin, F. Descotes, S. Saez, F. Spyratos

**Affiliations:** CIS Bio International, Division In Vitro Technologies, Bagnols-sur-CÃ¨ze, France.

## Abstract

In a study involving 50 breast cancer tumours, we compared two oestrogen receptor (ER) detection methods developed by us--a microplate immunoenzymometric assay (EIA96) and an immunohistochemistry kit (HistoCIS-ER)--with the radioligand assay (RLA), the Abbott immunoenzymometric assay ER-EIA and the reverse transcriptase polymerase chain reaction technique (RT-PCR). Among the three ER protein cytosolic assays (EIA96, ER-EIA and RLA), the two EIAs showed the best agreement (y = 1.086x - 7.840; r2 = 0.876). At the calculated optimal cut-off values (8 and 14 fmol mg(-1) protein for EIA96 and RLA respectively), EIA96 was more sensitive than RLA (0.94 for EIA96, 0.88 for RLA), but slightly less specific (0.82 for EIA96, 0.94 for RLA). The Cox logistical regression model applied to EIA96, RLA and RT-PCR showed that EIA96 discriminated the best between ER-EIA+ and ER-EIA- samples. The RT-PCR technique and HistoCIS-ER both had a positivity-negativity concordance of 86% with EIA96.


British Journal of Cancer (1997) 76(4), 519–525
© 1997 Cancer Research Campaign

### Comparison of a new microplate oestrogen receptor (ER) enzyme immunoassay with other ER detection methods

**V Delage[1], S Deytieux[2], V Le Doussal[2], F Degorce[1], L Bellanger[1], K Hacene[2], P Seguin[1], F Descotes[3], S Saez[3] and F Spyratos[2]**

[1]CIS Bio International, Division In Vitro Technologies, BP 175, 30203 Bagnols-sur-Cèze cedex, France; [2]Centre René Huguenin, 35, rue Dailly, 92211 Saint-Cloud, France; [3]Centre Hospitalier Lyon-Sud, Service de Techniques Nucléaires et Biophysiques, Laboratoire de Radioanalyse, 69495 Pierre Bénite cedex, France

**Summary** In a study involving 50 breast cancer tumours, we compared two oestrogen receptor (ER) detection methods developed by us – a microplate immunoenzymometric assay (EIA96) and an immunohistochemistry kit (HistoCIS-ER) – with the radioligand assay (RLA), the Abbott immunoenzymometric assay ER-EIA and the reverse transcriptase polymerase chain reaction technique (RT-PCR). Among the three ER protein cytosolic assays (EIA96, ER-EIA and RLA), the two EIAs showed the best agreement ($y = 1.086x - 7.840$; $r^2 = 0.876$). At the calculated optimal cut-off values (8 and 14 fmol mg$^{-1}$ protein for EIA96 and RLA respectively), EIA96 was more sensitive than RLA (0.94 for EIA96, 0.88 for RLA), but slightly less specific (0.82 for EIA96, 0.94 for RLA). The Cox logistical regression model applied to EIA96, RLA and RT-PCR showed that EIA96 discriminated the best between ER-EIA$^+$ and ER-EIA$^-$ samples. The RT-PCR technique and HistoCIS-ER both had a positivity–negativity concordance of 86% with EIA96.

**Keywords:** steroid receptor; enzyme immunoassay; radioligand assay; immunohistochemistry; reverse transcriptase polymerase chain reaction

The determination of the oestrogen receptor (ER) content of a breast tumour is of clinical interest in two respects. First, the presence of ER is an independent prognostic factor (Knight et al, 1977); the prognosis of patients with ER-positive tumours is more favourable than that of patients with ER-negative tumours (Horwitz et al, 1975). On the other hand, the ER/progesterone receptor (PgR) status is a predictor of response to hormone therapy: 70–80% of patients with ER$^+$/PgR$^+$ breast tumour biopsies respond to hormonal treatment compared with 10% of patients with ER$^-$/PgR$^-$ tumours (McGuire et al, 1991).

The oestrogen receptor is a 66-kDa ligand-regulated transcriptional factor that is encoded by a gene composed of eight exons (Green et al, 1986). Among the six domains of ER (A–F), the C region is responsible for DNA binding and the E domain holds the ligand-binding function. Many mutations and variants of the ER mRNA have been described in mammary tumours and in breast cancer cell lines (Sluyser, 1995), as well as in normal breast tissue (Pfeffer et al, 1995). The vast majority of these mutations arises from an alternative splicing of the transcript, leading to the deletion of one or more exons. This phenomenon is of variable extent and has been described for exons 2, 3, 4, 5 and 7. The proteins encoded by aberrantly spliced mRNAs are truncated or internally deleted. The expression of variant receptors in heterologous systems reveals dominant-positive ERs that are transcriptionally active in the absence of hormone (the case of ERΔE5, an ER encoded by an exon 5 deleted mRNA; Fuqua et al, 1991), whereas ERΔE7 is dominant negative as it is transcriptionally inactive and prevents the action of the wild-type receptor (Wang et al, 1991; Fuqua et al, 1992). Apart from these splicing variants, ponctual mutations and nucleotide insertions have also been described (Sluyser, 1995). Some variant receptors have also been found in normal tissues (Hoshino et al, 1995; Pfeffer et al, 1995) raising the question whether the relative levels of variant ERs in tissues may be important in relation to loss of hormone dependence.

The ER assay can be carried out at the protein level as well as at the mRNA level. In the case of protein detection, two kinds of techniques are widely used: quantitation of ER in cytosol extracts of tumour tissue and immunohistochemistry on tissue sections.

The receptor assay in cytosol extracts can be made using two techniques based on different principles. The reference method is the radioligand assay (RLA), which detects the ligand-binding function by measuring the number of specific binding sites and their affinity for the ligand. An enzyme immunoassay (EIA) detects the physical presence of ERs when the epitopes recognized by the antibodies remain intact; these receptors can therefore be functionally inactive. The ER-EIA kit developed by the Abbott Laboratories recognizes epitopes localized in domains D and E of ER (regions 250–302 and 463–526; Leclercq et al, 1986).

Anti-ER monoclonal antibodies are also used in immunohistochemistry to visualize the tissue distribution and subcellular localization of the receptor on tissue sections. The percentage of stained cells and the staining intensity allow a semiquantification. The information provided by immunohistochemistry is complementary to the results obtained with quantitative methods carried out on cytosol extracts.

*Received 29 October 1996*
*Revised 31 January 1997*
*Accepted 17 February 1997*

*Correspondence to:* V Delage

We have developed an immunoenzymometric assay for ER, EIA96 (Delage et al, 1996), and an immunohistochemistry kit, HistoCIS-ER both of which recognize the B region of the receptor. The aim of the present study was to compare EIA96 with the other quantitative detection techniques of the ER protein (RLA, ER-EIA) and with the RT-PCR applied to the mRNA region that codes for the C domain of the receptor as well as with the immunohistochemistry method.

### MATERIALS AND METHODS

### Sample collection

Receptors were measured in breast adenocarcinoma tissue biopsies. The samples were obtained by surgical removal and immediately frozen in liquid nitrogen before transferral to the laboratory. None of the patients in this study had received chemo- or radiotherapy before surgical treatment. Each piece was histologically examined by the pathologist in order to check the presence and appearance of tumoral tissue.

### Reagents

We used the following buffers: phosphate-buffered saline (PBS): disodium hydrogen phosphate/potassium dihydrogen phosphate 50 mmol $l^{-1}$, sodium chloride 150 mmol $l^{-1}$, pH 7.4; TEM buffer: *Tris*-HCl 10 mmol $l^{-1}$, EDTA 1.5 mmol $l^{-1}$, sodium molybdate 5 mmol $l^{-1}$, monothioglycerol 0.1 ml $l^{-1}$, pH 7.4 (TEGM buffer was made as TEM buffer by replacing monothioglycerol by 100 ml $l^{-1}$ glycerol); citrate buffer: citric acid monohydrate 100 mmol $l^{-1}$, trisodium citrate dihydrate 100 mmol $l^{-1}$, pH 6.0; buffer A: disodium hydrogen phosphate/sodium dihydrogen phosphate 50 mmol $l^{-1}$, potassium chloride 400 mmol $l^{-1}$, pH 7.0; buffer B: disodium hydrogen phosphate/sodium dihydrogen phosphate 50 mmol $l^{-1}$, pH 8.0; xylene cyanol blue: xylene cyanol blue 0.25%, bromophenol blue 0.25%, EDTA 1 mmol $l^{-1}$, glycerol 50%, pH 8.0; buffer 67: *Tris*-HCl 670 mmol $l^{-1}$, ammonium sulphate 166 mmol $l^{-1}$, magnesium chloride 67 mmol $l^{-1}$, pH 8.8; buffer 27: *Tris*-HCl 670 mmol $l^{-1}$, ammonium sulphate 166 mmol $l^{-1}$, magnesium chloride 27 mmol $l^{-1}$, pH 8.8. Buffers 10 × TAE and 10 × TBE were purchased from Interchim (Asnières, France).

17β-[2,4,6,7-$^3$H]oestradiol, sp. act. $3.3 \times 10^{15}$ –$3.9 \times 10^{15}$ Bq $mol^{-1}$, was purchased from Amersham (Les Ulis, France). Inert steroids and diethylstilboestrol were obtained from Roussel-Uclaf (Romainville, France). The radiolabelled steroid was purified by paper chromatography, eluted with absolute ethanol and stored under nitrogen in amber vials at –20°C until use. Inert steroids were prepared as 1 mmol $l^{-1}$ solutions and stored at 4°C.

### Reverse transcriptase polymerase chain reaction (RT-PCR)

#### *RNA extraction*

Frozen tumour specimens were homogenized using a Polytron 2000 (Bioblock Scientific, Illkirch Graffenstaden, France) in Trizol (0.1 g per 1 ml; Life Technologies, Eragny, France). After a 5-min incubation at room temperature, 0.2 volume of chloroform was added and the tubes were shaken. After a 2- to 3-min incubation at room temperature, a 20-min centrifugation at 12 000 ***g*** at 4°C was performed using a TL-100.4 rotor in a TL-100 centrifuge (Beckman Instruments, Gagny, France). The aqueous superior phase was transferred to a new tube and one volume of isopropanol was added. After a 16- to 18-h incubation at –20°C, a 20-min centrifugation at 20 000 ***g*** at 4°C was performed. The supernatant was discarded and the pellet washed with 1 ml of a 75% ethanol solution. After a 15-min centrifugation at 12 000 ***g*** at 4°C, the pellet was dried and dissolved in 50–100 μl of sterile diethylpyrocarbonate (DEPC)-treated water. Samples were heated to 60°C for 10 min.

The RNA concentration was determined spectrometrically (Spectronic Genesys 5, Bioblock Scientific) using the absorbance at 260 nm of 5 μl of RNA diluted in 500 μl of DEPC water. The RNA concentration was adjusted to 1 μg $μl^{-1}$ in DEPC water. The absence of protein contamination was checked using the ratio $A_{260}/A_{280}$, which had to be higher than 1.6. Samples were stored at –80°C.

An aliquot (1 μl) of RNA was added to 10 μl of water and 1 μl of xylene cyanol blue. Samples were applied on a 2% agarose gel and electrophoresed for 15 min in 1 × TAE buffer at 100 V (Super-Sub HE100, Hoefer, Pharmacia Biotech, Uppsala, Sweden). The gel was stained with ethidium bromide. A good RNA extraction produced three bands (28S, 18S, 4S) of decreasing intensity. Deteriorated samples were eliminated.

#### *Reverse transcription*

An aliquot (1 μl) of RNA (1 μg $μl^{-1}$) or 1 μl of sterile water (control) was mixed with 2 μl of a 10 mmol $l^{-1}$ dNTP mix (Life Technologies), 1 μl of 100 μmol $l^{-1}$ PdN6 random hexamer (Boehringer Mannheim France SA, Meylan, France), 2 μl of buffer 67 and 12 μl of sterile water. After an initial incubation at 70°C for 5 min and a brief centrifugation, samples were put on ice. An aliquot (1 μl) of human placental ribonuclease inhibitor (120 U $μl^{-1}$, Amersham) and 1 μl of murine moloney leukaemia virus (MuML V) reverse transcriptase (200 U $μl^{-1}$, Life Technologies) was added. Reverse transcription was allowed to proceed for 30 min at 42°C and another 5 min at 75°C. Samples were put on ice after a brief centrifugation. A total of 480 μl of DEPC water was added and cDNA samples were stored at –80°C.

#### *Polymerase chain reaction*

An aliquot (10 μl) of cDNA was added to 2 μl of 10 mmol $l^{-1}$ dNTP mix, 1 μl of each of the 50 pmol $μl^{-1}$ ER primers (no. 1 and no. 2, Genosys Biotechnologies, The Woodlands, TX, USA), 2 μl of each of the 50 pmol $μl^{-1}$ $β_2$-microglobulin primers (no. 3 and no. 4, Genosys Biotechnologies), 10 μl of buffer 27, 0.5 μl of *Taq* DNA polymerase (2.5 U $μl^{-1}$, Life Technologies), 71.5 μl of sterile distilled water and 100 μl of vaselin oil (Prolabo, Paris, France). The sense ER primer no. 1 (5′-ACTCGCTACTGTGCAGTGTG-CAATG-3′) corresponds to ER cDNA 544–568 (Greene et al, 1986). ER primer no. 2 (5′-CCTCTTCGGTCTTTTCGTATCCCAC-3′) represents the antisense strand of the ER cDNA sequence 758–782 (Greene et al, 1986). The sense $β_2$-microglobulin primer no. 3 (5′-CATCCAGCGTACTCCAAAGA-3′) corresponds to $β_2$-microglobulin cDNA 97–116 (Güssow et al, 1987). $β_2$-Microglobulin primer no. 4 (5′-GACAAGTCTGAATGCTCCAC-3′) represents the antisense strand of the $β_2$-microglobulin cDNA sequence 242–261 (Güssow et al, 1987). After an initial denaturation at 94°C for 30 s, each cycle of amplification consisted in a 50-s denaturation at 94°C, followed by 50 s of annealing (58°C) and 20 s of extension (72°C) steps. After 35 cycles, the final product was extended for 10 min (70°C; Temp-tronic Thermolyne, Bioblock Scientific). The following controls were included: (1) T47D cell line (ER+, $β_2$-microglobulin+); (2) HT1080 cell line (ER-, $β_2$-microglobulin+); (3) sterile water (PCR–); (4) RT-control product.

© Cancer Research Campaign 1997

**Table 1** Variable distribution (ER protein assays)

| | Minimum | Q25 | Median | Q75 | Maximum |
|---|---|---|---|---|---|
| RLA | 0 | 3.6 | 23.5 | 57.9 | 645 |
| EIA96 | 0 | 4.1 | 25.0 | 54.6 | 478 |
| ER-EIA | 1 | 10.2 | 28.5 | 74.5 | 330 |

**Table 2** Statistics and tests at the optimal cut-off value of each method

| Statistics at the optimal cut-off value | RT-PCR | RLA | EIA96 |
|---|---|---|---|
| Optimal cut-off value | 0.21 | 14 | 8 |
| Optimal $\chi^2$ | 20.06 | 31.43 | 30.01 |
| Corrected *P*-value | $9.9 \times 10^{-4}$ | $6.6 \times 10^{-5}$ | $7.3 \times 10^{-5}$ |
| *K* | 0.62 | 0.79 | 0.77 |
| Standard error | 0.12 | 0.09 | 0.10 |
| *P*-value | $2.4 \times 10^{-7}$ | $< 10^{-8}$ | $< 10^{-8}$ |
| $P^+$ | 0.89 | 0.92 | 0.93 |
| $P^-$ | 0.73 | 0.86 | 0.85 |
| Area under ROC | 0.793 | 0.910 | 0.881 |
| Standard error | 0.063 | 0.041 | 0.052 |
| Sensitivity | 0.94 | 0.88 | 0.94 |
| Specificity | 0.65 | 0.94 | 0.82 |
| LR | 2.66 | 14.94 | 5.32 |
| $V_{pp}$ | 0.84 | 0.97 | 0.91 |
| $V_{np}$ | 0.85 | 0.80 | 0.88 |
| % CC | 84 | 90 | 90 |

LR, likelihood ratio; $V_{pp}$, positive predictive value; $V_{np}$, negative predictive value; % CC, percentage of correctly classified tumours.

**Figure 1** Correlation between EIA96 and ER-EIA, EIA96 and RLA, and RLA and ER-EIA on 50 cytosols of mammary tumours. A one-degree polynomial curve fit was used. $n = 50$; ···O···, RLA = 1.173 × ER-EIA – 13.398, $r^2 = 0.759$; -■-, EIA96 = 1.086 × ER-EIA – 7.840, $r^2 = 0.876$; --✱--, EIA96 = 0.777 × RLA + 13.170, $r^2 = 0.814$

An aliquot (100 µl) of the PCR product was added to 10 µl of xylene cyanol blue. Of this mixture, 20 µl was applied on a 2% agarose gel and electrophoresed in 1 × TAE buffer for 1 h at 100 V. The gel was stained with ethidium bromide. UV visualization allowed checking of the PCR efficiency (integrity of $\beta_2$-microglobulin and of each control).

An aliquot (20 µl) of the PCR product–xylene cyanol blue mixture was then applied on a 7% polyacrylamide gel prepared as follows: 10.5 ml of 40% acrylamide (38/2, Appligene, Illkirch Graffenstaden, France), 6 ml of 10 × TBE, 43.5 ml of sterile distilled water, 360 µl of ammonium peroxidisulfate (E Merck, Darmstadt, Germany), 45 µl of Temed (Sigma Chemical, St Louis, MO, USA). Samples were electrophoresed for 16 to 18 h at 4°C (20 V). After ethidium bromide coloration, the density of each ER and $\beta_2$-microglobulin band was estimated using the BIO-PROFIL system (BIOID logiciel, Vilber Lourmat, Torcy, France). For each sample, the R ratio was calculated: R = density of ER band–density of $\beta_2$-microglobulin band.

### Radioligand method and oestrogen receptor immunoenzymometric assays

### *Cytosol preparation*

An aliquot (0.2–0.3 g) of each frozen tumour specimen was homogenized for 15 s using a Polytron 2000 (Bioblock Scientific) in cold TEGM buffer (10 ml g$^{-1}$ tumour). The cytosol was obtained by ultracentrifuging the homogenate at 105 000 ***g*** for 1 h at 4°C (TL-100.4 rotor in a TL-100 ultracentrifuge, Beckman Instruments) and immediately stored in liquid nitrogen.

### *Radioligand method*

Cytosol aliquots were distributed in 96-well microtitre plates. The presence and the number of ER binding sites were determined by the RLA, as described previously (Delage et al, 1996), and according to the recommendations of the European Organization for the Research and Treatment of Cancer (EORTC Breast Cancer Cooperative Group, 1980). The results were expressed as fmol bound oestradiol mg$^{-1}$ of cytosolic protein.

### *Microtitre plate EIA96 assay protocol*

All steps were carried out at 4°C in 96-well microtitre plates (Greiner-Labortechnik, Frickenhausen, Germany), as described previously (Delage et al, 1996). The mouse monoclonal antibodies involved in EIA96 were B10 (Ali et al, 1993), directed against amino acids 151–165 of the B domain of the human ER (Krust et al, 1986), and AER314 (Bioprobe, Amstelveen, The Netherlands; Abbondanza et al, 1993), directed against an epitope distinct from B10 in the B domain (region 121–168). Calibrated solutions of recombinant human ER expressed in yeast strain TGY14 were used as standards (Metzger et al, 1988). The results were expressed as fmol ER mg$^{-1}$ of cytosolic protein.

### *Abbott ER-EIA*

ER was assayed with the Abbott ER-EIA monoclonal kit (Laboratories Abbott, Rungis, France), according to the manufacturer's instructions. ER concentration was expressed as fmol mg$^{-1}$ of cytosolic protein.

### Protein concentration

The protein concentration of cytosols was determined using the BCA protein assay kit (Pierce Europe, Oud Beijerland, The Netherlands), according to the manufacturer's instructions. Bovine serum albumin supplied in the kit was used as the calibrator.

© Cancer Research Campaign 1997

### Immunohistochemistry

For each tumour, one or two representative paraffin blocks were chosen, with adjacent non-tumoral tissue (internal control). Four-micrometre tissue sections were mounted onto APES-pretreated slides (3-aminopropylethoxysilane, Sigma Chimie, Saint Quentin Fallavier, France) and dried at 55°C overnight. The slides were deparaffinized for 20 min in toluene, 2 × 5 min in 100% ethanol, and washed for 5 min in tap water. They were then put in citrate buffer and heated in a microwave oven for 4 × 5 min. After each 5-min cycle, the level was topped up with distilled water. Slides were left to cool at room temperature for 20 min, incubated for another 20 min in hydrogen peroxide 2% in methanol, and washed for 3 min in tap water. The sections were then incubated at room temperature for 10 min with a blocking reagent [bovine serum albumin (BSA) 1% in PBS]. Excess reagent was discarded and the slides were incubated with primary antibody (B10) diluted in PBS/BSA (2 μg $ml^{-1}$) at room temperature for 1 h in a humidified chamber. They were then washed for 2 × 5 min in PBS, incubated at room temperature with a biotinylated goat anti-mouse antibody (StreptABComplex Duet, Dako, Trappes, France), rinsed for 2 × 3 min in PBS and then incubated for another 30 min at room temperature in the chamber with the streptavidin–biotin–peroxidase complex from the same kit. The slides were rinsed for 2 × 3 min in PBS, and finally incubated for 10 min with a DAB solution (diaminobenzidine, Biosys, Compiègne, France). After one wash with tap water, the slides were counterstained with Hemalun (E Merck) for 10 s, and rinsed in tap water. The slides were mounted in Aquatex (E Merck). A semiquantitative evaluation was carried out by the pathologist to estimate the percentage of positive cells and the staining intensity. A section was considered $ER^+$ if more than 10% of cells were stained.

### Data analysis

Taking ER-EIA as the reference technique (cut-off value = 15 fmol $mg^{-1}$ protein), the optimal cut-off value of the three other quantitative techniques ($X$ = EIA96, RLA or RT-PCR) was determined by considering each of the distinct values obtained for $X$ as the cut-off value and by calculating the associated $\chi^2$ with one degree of freedom. The value associated with the maximum $\chi^2$ was taken as the cut-off value. The associated $P$-value was corrected following the Hilsenbeck and Clark (1996) method, by discarding 1% of the low and high values of $X$. ER-EIA$^+$/$X^+$ were

**Figure 2** Relationship between RT-PCR and EIA96 on 50 mammary tumours. The calculated optimal cut-off values are indicated for each method then considered as true positive (TP), ER-EIA$^-$/$X^-$ as true negative (TN), ER-EIA$^-$/$X^+$ as false positive (FP) and ER-EIA$^+$/$X^-$ as false negative (FN). The sensitivity and the specificity were the true positive rate [TPR = TP/(TP + FN)] and the true negative rate [TNR = TN/(TN + FP)] respectively. The positive predictive value of an $X$ technique [$V_{pp}$ = TP/(TP + FP)] was the probability that a tumour sample assayed ER+ by the $X$ technique was really $ER^+$. On the other hand, the negative predictive value [$V_{np}$ = TN/(TN + FN)] was the probability that a tumour sample assayed $ER^-$ by the $X$ technique was really $ER^-$. The likelihood ratio (LR) was equal to sensitivity/(1 – specificity). The receiver-operating characteristic (ROC) curve, which was plotted as sensitivity against (1 – specificity), gave the discriminatory capacity of a test. The area under the ROC curve showed the probability that a random $ER^+$ tumour had been correctly classified.

The agreement between each of the three methods and ER-EIA was evaluated using the agreement coefficient $K$, with its standard error and the average of positive and negative agreements ($P^+$ and $P^-$ respectively).

A one-degree polynomial curve fit was used to correlate the data obtained with the methods quantifying the ER protein (EIA96, ER-EIA, RLA).

The Cox (1970) logistical regression model was applied to determine which technique among RLA, EIA96 and RT-PCR best discriminated ER-EIA$^+$ from ER-EIA$^-$ samples. The

**Table 3** Samples with at least one discrepant value in one of the five methods

| Tumour | EIA96 (fmol mg$^{-1}$) | ER-EIA (fmol mg$^{-1}$) | RLA (fmol mg$^{-1}$) | RT-PCR | HistoCIS-ER |
|---|---|---|---|---|---|
| 7 | 34.9 | 38.0 | **0.0** | 0.61 | 80% |
| 12 | **24.6** | 15.0 | 14.0 | 0.20 | 0% |
| 13 | 2.3 | 2.0 | 3.2 | **0.38** | 0% |
| 16 | 16.1 | 18.0 | **5.3** | 0.32 | 80% |
| 18 | 0.6 | 2.0 | 0.0 | **0.84** | **100%** |
| 27 | **22.6** | 13.0 | 0.0 | 0.04 | 0% |
| 37 | **4.0** | 26.0 | 30.0 | **0.16** | 50% |
| 46 | **5.4** | **10.0** | 112.4 | 0.55 | 80% |
| 50 | 21.1 | 23.0 | 14.0 | **0.07** | 90% |
| 51 | 4.0 | 2.0 | 3.6 | **0.38** | **20%** |
| 53 | **0.0** | 43.0 | 17.4 | 0.33 | 40% |

Discrepant values are indicated in bold.

© Cancer Research Campaign 1997

Lemshow–Hosmer test (Lemshow and Hosmer, 1982), the Harrel c index (Harrel et al, 1982) and the Goodman–Kruskal correlation index allowed checking to see if the model used was appropriate.

### RESULTS

The oestrogen receptor was detected in 50 mammary tumours using five different methods: determination of the ER protein concentration in cytosolic extracts using the radioligand binding assay (RLA) and two immunoenzymometric techniques (EIA96, ER-EIA), quantification by RT-PCR of the ER mRNA region coding for domain C, immunohistochemical detection of the receptor on sections with the B10 antibody (HistoCIS-ER).

### Assay of ER protein (RLA, EIA96, ER-EIA)

*Variable distribution*

Table 1 lists the minimum, maximum, lower (Q25) and upper (Q75) quartiles, and median values.

*Determination of optimal cut-off values*

Considering the maximum $\chi^2$ values, the cut-off values of EIA96 and RLA were 8 fmol $mg^{-1}$ and 14 fmol $mg^{-1}$ respectively (Table 2). At the optimal cut-off value, EIA96 and RLA displayed a substantial agreement with ER-EIA ($K$ = 0.77 and 0.79 respectively) and discriminated well between $ER^+$ and $ER^-$ tumours (area under ROC = 0.881 and 0.910 respectively). Furthermore, EIA96 was more sensitive than RLA at the optimal cut-off value (0.94 for EIA96, 0.88 for RLA), but slightly less specific (0.82 for EIA96, 0.94 for RLA).

*Correlations*

The two immunoenzymometric techniques, EIA96 and ER-EIA, showed the best correlation (Figure 1). Seven samples had at least one discrepant value in one of the three methods (Table 3, samples 7, 12, 16, 27, 37, 46, 53).

### Quantification of ER mRNA (RT-PCR)

For each tumour, the density of the ER band was divided by the density of the $\beta_2$-microglobulin band.

**Table 4** Cox logistical regression model

| $P_i$ | Observed ER-EIA$^+$ | Observed ER-EIA$^-$ | Predicted ER-EIA$^+$ | Predicted ER -EIA$^-$ |
|---|---|---|---|---|
| [0.0;0.1] | 0 | 0 | 0.00 | 0.00 |
| [0.1;0.2] | 1 | 8 | 1.45 | 7.55 |
| [0.2;0.3] | 1 | 5 | 1.31 | 4.69 |
| [0.3;0.4] | 1 | 1 | 0.69 | 1.31 |
| [0.4;0.5] | 1 | 0 | 0.47 | 0.53 |
| [0.5;0.6] | 1 | 0 | 0.57 | 0.43 |
| [0.6;0.7] | 2 | 0 | 1.27 | 0.73 |
| [0.7;0.8] | 2 | 1 | 2.25 | 0.75 |
| [0.8;0.9] | 3 | 2 | 4.21 | 0.79 |
| [0.9;1.0] | 21 | 0 | 20.76 | 0.24 |
| Total | 33 | 17 | 33 | 17 |

*Determination of the optimal cut-off value*

An optimal cut-off value of 0.21 was established (Table 2). At this value, the agreement between RT-PCR and ER-EIA was moderate ($K$ = 0.62); furthermore, the RT-PCR discriminatory ability was lower than those of EIA96 and RLA (area under ROC = 0.793). Although the RT-PCR sensitivity was identical to that of EIA96 at the cut-off value, its specificity was significantly lower.

*Comparison with EIA96*

Figure 2 shows the relation between the RT-PCR status and the concentration found by EIA96. Seven samples had discrepant results (14%): two tumours were RT-PCR$^-$/EIA96$^+$ (Table 3, tumours 27, 50) and five were RT-PCR$^+$/EIA96$^-$ (Table 3, tumours 13, 18, 46, 51, 53).

*Cox logistical regression model*

The Cox logistical regression model applied to the 50 tumours showed that, among the three techniques (EIA96, RLA, RT-PCR), EIA96 best discriminated between ER-EIA$^+$ and ER-EIA$^-$ samples (insertion $\chi^2$ associated $P$-value fixed at 5%).

The model also allowed the calculation of the $P_i$ probabilities for each tumour according to the following relationship

$$P_i = \text{prob(ER} - \text{EIA +/EIA96)} = \frac{\exp(b_0 + b_1 \times \text{EIA96})}{1 + \exp(b_0 + b_1 \times \text{EIA96})}$$

with $b_0 = -1.832$ (confidence interval = [–3.170; –0.495]) and $b_1$ = 0.133 [confidence interval = (0.052; 0.214), Table 4] and were then classified in 0.10-long intervals. There was a good agreement between the number of predicted ER-EIA$^+$ values and the number of observed ER-EIA + values in each interval ($y$ (number of predicted ER-EIA$^+$ values) = 0.992$x$ (number of observed ER-EIA$^+$ values) + 0.026, $r^2$ = 0.992). A Lemshow–Hosmer statistic of 5.991 was found ($P$ = 0.648 with eight degrees of freedom), indicating a good fitting of the model with the data.

The probability for a random sample that the observed and predicted values are in agreement was estimated by a Harrel c index of 0.922 (standard error = 0.039). A Goodman–Kruskal correlation index of 0.843 was calculated from c.

**Figure 3** Relationship between the percentage of stained cells (HistoCIS-ER) and EIA96 on 50 mammary tumours. The calculated optimal cut-off value of EIA96 is indicated

© Cancer Research Campaign 1997

### Immunohistochemistry (HistoCIS-ER)

The B10 antibody used for immunohistochemical detection is the same as the EIA96 solid-phase antibody. The staining of the cells was intranuclear. Except in some altered areas, non-specific staining was not observed. Figure 3 shows the relation between the HistoCIS-ER status and the concentration found by EIA96. Seven samples had discrepant results (14%): two tumours were HistoCIS-ER$^-$/EIA96$^+$ (Table 3, tumours 12, 27) and five were HistoCIS-ER$^+$/EIA96$^-$ (Table 3, tumours 18, 37, 46, 51, 53).

### DISCUSSION

We developed a microtitre plate immunoenzymometric assay of the oestrogen receptor, EIA96, using two antibodies directed against the B domain of ER (Delage et al, 1996). This study is a comparison of EIA96 with four other detection techniques for the receptor, involving 50 mammary tumours. The five methods compared can be classified in two groups: strictly quantitative assays of ER, carried out on the cytosolic fraction (EIA96, ER-EIA, RLA), and detection techniques for either the ER protein on tissue sections (HistoCIS-ER) or for a region of the receptor mRNA (RT-PCR).

The comparison of EIA96 with the two other assays carried out on the cytosolic fraction (RLA, ER-EIA) shows that the best-correlated techniques are the immunoenzymometric methods, EIA96 and ER-EIA. Assuming that ER-EIA is the reference method, EIA96 discriminates ER$^+$ from ER$^-$ samples better than RLA does. EIA96 and ER-EIA rely on the same principle, i.e. the detection of the receptor presence by immunological recognition, very different from the RLA principle, which measures the integrity of the hormone-binding function. In spite of these methodological differences, EIA96 and RLA behave similarly at the calculated optimal cut-off values: the agreement between the two assays and ER-EIA is substantial; EIA96 is slightly less specific but more sensitive than the RLA technique at the optimal cut-off value. The observed agreement between EIA96 and the most widely used assays (RLA and ER-EIA) argues in favour of the reliability of EIA96 results, which is very important from a clinical point of view.

The comparison between EIA96 and HistoCIS-ER or RT-PCR shows a positivity–negativity agreement of 86% in both cases. Although HistoCIS-ER is carried out on tissue sections instead of cytosols, as is the case for EIA96, this technique also detects the B domain of ER through B10, the EIA96 solid-phase antibody. Concerning RT-PCR, many events can generate discrepant results with EIA96. This technique measures a very different entity, i.e. the mRNA region that codes for the C domain of the receptor. Some discrepant results arise from the messenger lability when the protein is still present or, in contrast, from a dysfunction of the protein synthesis, the mRNA subsisting without being translated. Nevertheless, the RT-PCR technique optimization allowed a significant agreement with EIA96 to be reached.

Eleven tumours show at least one discrepant value in one of the five compared techniques. Samples 18 and, to a lesser extent, 51 have negative results with the three cytosolic quantitative assays of ER (EIA96, ER-EIA, RLA). On the other hand, the receptor detection on sections by HistoCIS-ER and the amplification by RT-PCR of an mRNA region are positive. The hypothesis of false-positive results does not seem to be valid because of the specificity of these two techniques. Indeed, a non-specific hybridization of the primers seems very unlikely, considering their length; in addition the B10 antibody was described as exclusively ER specific by Ali et al (1993). Finally, the histological characteristics of these tumours cannot explain the discrepancies. The most likely hypothesis would be a sampling heterogeneity because of the fact that a large quantity of each tumour was necessary for the comparison of the five techniques.

The discrepancies of tumours 12, 13, 16, 27, 37 and 50 are weak, the values being quite close to the calculated optimal cut-off values. These cut-off values were determined with regard to ER-EIA as a reference technique with a limited number of tumours (50). A clinical study involving a larger number of samples would allow the calculation of cut-off values with a better clinical significance than the technical cut-off values determined in the present work.

Tumours 7, 46 and 53 show a wide discrepancy in one of the values obtained with the assays carried out on the cytosolic fraction. These discrepancies cannot arise from a sampling heterogeneity, because the three techniques were carried out on the same cytosol, which was aliquoted immediately after preparation. The histological data of these tumours show no atypical characteristics. In addition, a new definition of the cut-off values would not allow correction of the discrepancies. Thus, the origin of these discrepant values remains unexplained.

The clinical importance of such discrepancies will have to be evaluated on a larger series of tumours. Nevertheless, this study confirms the results obtained in a previous work that compared EIA96 with the other cytosolic assays of ER (RLA and ER-EIA; Delage et al, 1996), and completes them by underlining the agreement between EIA96 and other detection methods (HistoCIS-ER and RT-PCR).

### ABBREVIATIONS

EIA, enzyme immunoassay; EIA96, microtitre plate oestrogen receptor immunoassay; ER, oestrogen receptor; HRP, horseradish peroxidase; RLA, radioligand assay; PBS, phosphate-buffered saline; EORTC, European Organization for the Research and Treatment of Cancer; DAB, diaminobenzidine; APES, 3-aminopropyltriethoxysilane; DEPC, diethylpyrocarbonate; BSA, bovine serum albumin.

### ACKNOWLEDGEMENTS

We are grateful to Professor Pierre Chambon and Dr Daniel Metzger (Institut de Génétique et de Biologie Moléculaire et Cellulaire, Centre National de la Recherche Scientifique, Institut National de la Santé et de la Recherche Médicale, Strasbourg, France) for kindly providing the transformed yeast strain TGY14 (expressing the human oestrogen receptor) and B10 antibody. VD is supported by CIS Bio International under the auspices of the Association Nationale de la Recherche Technique. The authors would like to acknowledge Dr Henri Magdelénat and Dr Sylvie Chevillart from the Laboratoire de transfert (Institut Curie, Paris, France) for their help in RT-PCR assays. We also wish to thank the Ligue Nationale Française Contre le Cancer, Comité des Hauts-de-Seine et des Yvelines, for their financial support.

#### REFERENCES

Abbondanza C, de Falco A, Nigro V, Medici N, Armetta I, Molinari AM, Montcharmont B and Puca GA (1993) Characterization and epitope mapping

© Cancer Research Campaign 1997

of a new panel of monoclonal antibodies to estradiol receptor. *Steroids* **58**: 4–12

Ali S, Lutz Y, Bellocq J-P, Chenard-Neu M-P, Rouyer N and Metzger D (1993) Production and characterization of monoclonal antibodies recognising defined regions of the human oestrogen receptor. *Hybridoma* **12**: 391–405

Cox DR (1970) *Analysis of Binary Data*. Chapman & Hall: London

Delage V, Teulon J-M, Bellanger L, Seguin P, Descotes F and Saez S (1996) Microtiter plate immunoenzymometric assay for estrogen receptor. *Clin Chem* **42**: 1955–1960

EORTC breast cancer cooperative group (1980) Revision of the standards for the assessment of hormone receptors in human breast cancer. *Eur J Cancer* **16**: 1513–1515

Fuqua SAW, Fitzgerald SD, Chamness GC, Tandon AK, McDonnell DP, Nawaz Z, O'Malley BW and McGuire WL (1991) Variant human breast tumor estrogen receptor with constitutive transcriptional activity. *Cancer Res* **51**: 105–109

Fuqua SAW, Fitzgerald SD, Allred DC, Elledge RM, Nawaz Z, McDonnell DP, O'Malley BW, Greene GL and McGuire WL (1992) Inhibition of estrogen receptor action by a naturally occurring variant in human breast tumors. *Cancer Res* **52**: 483–486

Green S, Walter P, Kumar V, Krust A, Bornert J-M, Argos P and Chambon P (1986) Human oestrogen receptor cDNA: sequence, expression and homology to v-*erb-A*. *Nature* **320**: 134–139

Greene GL, Gilna P, Waterfield M, Baker A, Hort Y and Shine J (1986) Sequence and expression of human estrogen receptor complementary DNA. *Science* **231**: 1150–1154

Güssow D, Rein R, Ginjaar I, Hochstenbach F, Seemann G, Kottman A and Ploegh HL (1987) The human $\beta_2$-microglobulin gene. Primary structure and definition of the transcriptional unit. *J Immunol* **139**: 3132–3138

Harrel FE, Califf RM, Pryor DB, Lee KL and Rosati RA (1982) Evaluating the yield of medical tests. *J Am Med Assoc* **247**: 2543–2546

Hilsenbeck SG and Clark GM (1996) Practical p-value adjustment for optimally selected cutpoints. *Stat Med* **15**: 103–112

Horwitz KB, McGuire WL, Pearson OH and Segaloff A (1975) Predicting response to endocrine therapy in human breast cancer: a hypothesis. *Science* **189**: 726–727

Hoshino S, Inoue S, Hosoi T, Saito T, Ikegami A, Kaneki M, Ouchi Y and Orimo H (1995). Demonstration of isoforms of the estrogen receptor in the bone tissues and in osteoblastic cells. *Calcif Tissue Int* **57**: 466–468

Knight WA, Livingston RB, Gregory EJ and McGuire WL (1977) Estrogen receptor as an independent prognostic factor for early recurrence in breast cancer. *Cancer Res* **37**: 4669–4671

Krust A, Green S, Argos P, Kumar V, Walter P, Bornert J-M and Chambon P (1986) The chicken oestrogen receptor sequence: homology with *v-erbA* and the human oestrogen and glucocorticoid receptors. *EMBO J* **5**: 891–897

Leclercq G, Bojar H, Goussard J, Nicholson RI, Pichon M-F, Piffanelli A, Pousette A, Thorpe S and Lonsdorfer M (1986) Abbott monoclonal enzyme immunoassay measurement of estrogen receptors in human breast cancer: a European multicenter study. *Cancer Res* **46** (suppl): 4233s–4236s

Lemshow S and Hosmer DW Jr (1982) Review of goodness of fit statistics for use in the development of logistic regression models. *Am J Epidemiol* **115**: 92–106

McGuire WL, Chamness GC and Fuqua SAW (1991) Estrogen receptor variants in clinical breast cancer. *Mol Endocrinol* **5**: 1571–1577

Metzger D, White JH and Chambon P (1988) The human oestrogen receptor functions in yeast. *Nature* **334**: 31–36

Pfeffer U, Fecarotta E and Vidali G (1995) Coexpression of multiple estrogen receptor variant messenger RNAs in normal and neoplastic breast tissues and in MCF-7 cells. *Cancer Res* **55**: 2158–2165

Sluyser M (1995) Mutations in the estrogen receptor gene. *Human Mutat* **6**: 97–103

Wang Y and Miksicek RJ (1991) Identification of a dominant negative form of the human estrogen receptor. *Mol Endocrinol* **5**: 1707–1715

© Cancer Research Campaign 1997